# An Essay on Clinical Instruction

**Published:** 1835-01-01

**Authors:** 


*An Essay on Clinical Instruction.* By E. Ch. A. Louis, M.D. Physician to the Hospital of La Pitié. Translated by Peter Martin, M.R.C.S. London. 1834. 8vo. pp. 33.

This Essay is divided into two parts; the first treating "of the study of particular facts, or of diseases considered in an isolated manner;" and the second, of the "resumé of a clinical course, or an inquiry into general facts."

The former part contains some hints on the method of examining patients, and investigating the history of their ailments; but we shall pass them over, as they are sufficiently familiar to every well-educated practitioner. This reminds us that an able physician, our late excellent friend Dr. George Pearson, drew up *a plan for the examination of patients*, which was not only a valuable help to beginners, but might be consulted with advantage by many experienced physicians. How often do we see, that, for the want of a sufficiently searching investigation at the first visit, some important fact comes to light, after a fortnight's medication, which ought to have been known ere a single draught had been swallowed!

In the second part, M. Louis insists very strongly on the advantages of what he calls "the numerical method;" or, in other words, on the advantages resulting from classing symptoms, and post-mortem appearances, with strict reference to their relative frequency. Thus, he says,

"If there exists an exception to a general law, the most general possible under the point of view which we are now considering, I mean the just appreciation of the symptoms, how are we to know it, except by means of the numerical method? Rusty, viscous, semitransparent sputa from one of the most remarkable and constant symptoms of pneumonia, and are very rarely absent, at least when the disease attacks a person previously in good health, and of mature years. Still this symptom has been absent, and in the circumstances just indicated; but in what proportion of cases? What is the value of this exception? It is not known, the numerical method has not extended so far." (P. 23.)

This fact may be illustrated by an instance from MS. notes of Louis's Lectures.

In his lecture on Pneumonia, after analysing the facts, he arrives at the following conclusions :

"Of thirty-seven cases, seven proved fatal. The mean age of those who recovered was forty-seven, of those who died, sixty-three. In seventeen cases the superior lobe was affected, and the mean age of the individuals was fifty-four, in twenty cases the inferior lobe was affected, and the mean age was thirty-five. Of the fatal cases the superior lobe was affected in five, the inferior in two. In twenty-seven of the cases the individuals were previously in good health, and in one half of the cases, the local symptoms were preceded by general symptoms, not referrible to any local cause."

"Again with regard to erysipelas. There were observed at La Pitié during a certain time, twelve cases of erysipelas, all of which terminated well; eleven commenced with shivering; in five there were general symptoms preceding any local symptoms. In six cases the nose was first affected, in four the cheeks, and in two the eyelids."

M. Louis concludes by saying that he has not placed among the difficulties of observation those which are supposed to arise from the slowness of the education of the senses, because he thinks that the exercise of the judgment has been confounded with that of the senses, which are only its instruments; and he believes that the ear is not less fine or less perfect in a beginner, than in one who is finishing his studies; "nor in him who interprets ill the phenomena of auscultation, than those who are most skilled in this art. Both hear the same sounds in the same subjects," &c. p. 32. This celebrated physician is here clearly in the wrong; nothing is more certain than that a novice cannot hear the sounds which can be distinguished by a proficient in the use of the stethoscope; and, moreover, that by time, and a series of efforts, he may learn to hear. Dr. Wollaston mentions his having known several persons, who, though not otherwise deaf, were insensible to some acute sounds, as, for example, the chirrup of the cricket; now it appears to us as certain that many of these *surdastri* might have been cured by directing their attention to sounds of gradually increased acuteness, as it is that a flabby townsman may be taught to raise the maximum of his walk from five miles to fifty.

This little treatise will be very useful to the zealous student; it will not merely furnish him with many valuable hints, but what is of still greater importance, will supply him with ample materials for thinking.

